# Sizing the lung in dogs: the inspiratory capacity defines the tidal volume

**DOI:** 10.5935/0103-507X.20180028

**Published:** 2018

**Authors:** Pablo Alejandro Donati, Emiliano Gogniat, Matías Madorno, Juan Manuel Guevara, Eliana Carolina Guillemi, María del Carmen Lavalle, Francisco Patricio Scorza, Germán Federico Mayer, Pablo Oscar Rodriguez

**Affiliations:** 1 Unidade de Terapia Intensiva Veterinária UCIcoop - Buenos Aires, Argentina.; 2 Unidade de Terapia Intensiva, Hospital Italiano de Buenos Aires - Buenos Aires, Argentina.; 3 Instituto Tecnológico de Buenos Aires - Buenos Aires, Argentina.; 4 Unidade de Terapia Intensiva, Centro de Educación Médica e Investigaciones Clínicas - Buenos Aires, Argentina.

**Keywords:** Inspiratory capacity, Tidal volume, Lung, Dogs

## Abstract

**Objective:**

To evaluate a novel physiological approach for setting the tidal volume in mechanical ventilation according to inspiratory capacity, and to determine if it results in an appropriate mechanical and gas exchange measurements in healthy and critically ill dogs.

**Methods:**

Twenty healthy animals were included in the study to assess the tidal volume expressed as a percentage of inspiratory capacity. For inspiratory capacity measurement, the mechanical ventilator was set as follows: pressure control mode with 35cmH_2_O of inspired pressure and zero end-expiratory pressure for 5 seconds. Subsequently, the animals were randomized into four groups and ventilated with a tidal volume corresponding to the different percentages of inspiratory capacity. Subsequently, ten critically ill dogs were studied.

**Results:**

Healthy dogs ventilated with a tidal volume of 17% of the inspiratory capacity showed normal respiratory mechanics and presented expected PaCO_2_ values more frequently than the other groups. The respiratory system and transpulmonary driving pressure were significantly higher among the critically ill dogs but below 15 cmH_2_O in all cases.

**Conclusions:**

The tidal volume based on the inspiratory capacity of each animal has proven to be a useful and simple tool when setting ventilator parameters. A similar approach should also be evaluated in other species, including human beings, if we consider the potential limitations of tidal volume titration based on the calculated ideal body weight.

## INTRODUCTION

In engineering, the terms stress and strain are used to describe the microscopic response of a body to external loading. Strain is the relative change in size and shape, whereas stress is the internal tension. Thus, Chiumello et al. concluded that the stress and strain used in engineering have clinical equivalents in the lung and that both parameters are important determinants of ventilator-induced lung injury (VILI) risk.^(^^1^^)^ Thus, the lung stress is represented by the transpulmonary pressure difference between the end of expiration and the end of inspiration (ΔP_L_), whereas strain is characterized by the change in the volume with regard to the functional residual capacity (FRC).^(^^2^^)^

Stress and strain are nearly linearly related up to the total lung capacity (TLC), with stress equivalent to k x strain.^(^^3^^)^

The strain value required to reach inspiratory capacity (IC) (the limit of physical lung expansion) is very similar between species: 2 - 2.3 in mice,^(^^4^^-^^5^^)^ 2 - 3 in rats,^(^^5^^,^^6^^)^ 2.6 in pigs,^(^^7^^)^ 2.2 in humans^(^^7^^,^^8^^)^ and 2 - 2.2 in dogs.^(^^7^^)^ In humans, following the publication of the ARDS Network Study, the setting of tidal volume (VT) (one of the determinants of strain) during mechanical ventilation (MV) is currently based on the ideal body weight (calculated using height and sex), assuming a linear relationship between the latter and normal lung volumes regardless of other sources of variation, such as age and race or anthropometric measurements errors.^(^^9^^-^^11^^)^ A different approach based on the size of the lungs has been proposed. The FRC and TLC may vary widely in humans and dogs with lung injuries, as well as in obese humans.^(^^12^^-^^14^^)^ Because the determination of FRC is technically difficult, previous publications proposed the estimation of IC instead of the functional lung size.^(^^13^^,^^14^^)^ The selection of VT according to a percentage of the measured IC allows for a greater level of individualization, even across different species.

We hypothesize that setting the VT according to IC during MV would generate the appropriate mechanical and gas exchange measurements in healthy and critically ill dogs and may become a novel physiological approach for MV.

The objective of the study was to evaluate if setting the VT according to IC may result in an appropriate mechanical ventilation strategy.

## METHODS

The protocol (E/121) was approved by the Ethics Committee from the *Hospital Italiano de Buenos Aires*. The procedures were explained to the patient owners and their signed informed consent was obtained prior to intervention.

To determine the upper inflection points for airway pressure (PiflexSupAw) and transpulmonary pressure (PiflexSupTP), pressure-volume (P-V) curves were constructed in two healthy dogs as the first step. Then, twenty healthy animals requiring general anesthesia for surgical procedures were included in the study to assess the VT expressed as a percentage of IC associated with normal respiratory mechanics and the partial pressure of carbon dioxide (PaCO_2_). Finally, data from 10 critically ill hospitalized dogs referred to the veterinary Intensive Care Unit UCIcoop and ventilated with the VT value were also analyzed.

### Constructing pressure-volume curves in healthy dogs

The TLC was defined for healthy dogs as the lung volume during forced inflation at an airway pressure of 35cmH_2_O.^(^^15^^)^ To test this hypothesis, two healthy dogs that were submitted to ovariectomy or orchiectomy were evaluated to calculate P-V curves and determine the upper inflection points for airway pressure, PiflexSupAw and PiflexSupTP. The assessment of healthy condition consisted of a complete clinical evaluation, thoracic radiographs and arterial blood gas measurements. All animals were treated with an opioid drug (tramadol; dosage: 3mg/kg IV), supplemented with oxygen through a face mask and pharmacologically induced with propofol (dosage: 6 to 8mg/kg IV). An orotracheal intubation was performed using the larger diameter endotracheal tube estimated by palpation of the trachea and the tube cuff was inflated. Then, an esophageal balloon catheter was placed by measuring the length of the catheter such that the tip was placed at the lower third of the esophagus. The position of the balloon was confirmed by an airway occlusion test performed by reducing the sedation level and allowing the animal to spontaneously inspire. The presence of a negative deflection in the esophageal pressure curve during the test verified the correct position of the balloon. A continuous infusion of propofol was administered, titrating the dose to achieve an adequate sedation level according to a previously reported sedation scale.^(^^16^^)^ Then, the animals were ventilated using a VT of 15mL/kg (volume considered normal in canines)^(^^17^^)^ in volume control mode (VC-CMV). Thereafter, a neuromuscular blocking agent was administered (atracurium; dosage: 6µg/kg/min IV). To generate the P-V curve, we used the low flow insufflation technique. A VT of 3000mL was selected at a flow rate of 7L/m for the two dogs, and the maximum airway pressure alarm was set at 40cmH_2_O to stop inspiration at that pressure regardless of the delivered VT. The airway and esophageal pressure, as well as the volume and flow delivered by the ventilator, were recorded throughout the entire procedure using a respiratory mechanics monitor (FluxRewiew GrT CO_2_ Software, MBMed, Buenos Aires, Argentina). The respiratory system and lung P-V curve were drawn using an offline analysis that included the software for the monitor. To determine the upper inflection point of both P-V curves, we used the method described by Venegas et al,^(^^18^^)^ with a nonlinear approximation^(^^19^^)^ generated by statistics software.

From the P-V curve analysis of data obtained from the 2 healthy dogs, we obtained average PiflexSupAw and PiflexSupTP values of 34.75 and 18.11cmH_2_O, respectively.

### Mechanical ventilation in healthy dogs using different inspiratory capacity percentages

Healthy condition was assessed on the basis of a complete physical examination. Ketamine (dosage: 5 - 10mg/kg IV; Ketonal 50 - Richmond, Buenos Aires, Argentina) and diazepam (dosage: 0.5mg/kg IV - Diazepet Brouwer, Buenos Aires, Argentina) were used for anesthetic induction and maintenance. The animals were treated with an opioid drug (nalbuphine; 0.5 - 1mg/kg IM; Nalbufine 10 - Richmond, Buenos Aires, Argentina or tramadol; dosage: 3mg/kg IV; Algen 20 - Richmond, Buenos Aires, Argentina).

Endotracheal intubation and esophageal balloon placement were performed. The dogs were maintained under controlled MV using VC-CMV. For IC measurement, the mechanical ventilator was set as follows: pressure control mode (PC-CMV) with 35cmH_2_O of inspired pressure and zero end-expiratory pressure (ZEEP) for 5 seconds. The volume delivered by the mechanical ventilator under these conditions was recorded. Subsequently, using sealed envelopes, the animals were randomized into four groups and ventilated in a VC-CMV with a VT corresponding to the different percentages of IC as follows: Group 1: 13%, Group 2: 17%, Group 3: 21%, and Group 4: 25%. The selection of these IC percentage values was based on previously published data for human patients (15 and 28%)^(^^14^^,^^20^^,^^21^^)^ and from our own experience (unpublished information).

The other ventilation parameters were as follows: respiratory rate of 15 breaths per minute (normal for a resting dog);^(^^22^^)^ an inspiratory time of 1 second; positive end-expiratory pressure (PEEP) of 0cmH_2_O; and fraction of inspired oxygen (FIO_2_) of 100%.

A femoral artery puncture was performed after 10 minutes of MV to test the PaCO_2_. For this purpose, a blood gas and electrolytes analyzer was used (EPOC Analyzer, Alere). The airway and esophageal pressure values were recorded throughout the procedure using a FluxMed Respiratory Monitor (MBMed, Argentina). We used esophageal pressure as a surrogate for pleural pressure.^(^^23^^)^

The inspiratory and expiratory transpulmonary pressures were calculated by subtracting the inspiratory and expiratory esophageal pressures from the plateau pressure and PEEP, respectively.^(^^24^^)^ Hemodynamic stability was assured in all patients by monitoring of clinical parameters (heart rate, mucous membrane color, capillary refill time and temperature), systolic blood pressure using the noninvasive Doppler technique (Parks Electronical Doppler Model 811B, Perimed UK, Bury St Edmunds, UK) and pulse oximetry (measured in the tongue) (Oximax NPB-40; Nellcor^TM^ Puritan Bennett Inc. 710 Medtronic Parkway, Minneapolis, EUA)

### Mechanical ventilation in critically ill dogs

Ten critically ill dogs under MV due to medical reasons were studied. The anesthetic protocol was adjusted to the pathology for which MV was indicated. Endotracheal intubation and esophageal balloon placement were performed as previously described in this study. The critically ill animals were ventilated in VC-CMV mode. The IC was determined as previously described, and the VT was set as a percentage of the estimated IC. The selected percentage was the one considered the most appropriate based on the results observed in the 20 healthy dogs (17%). The respiratory rate was set between 15 and 25bpm according to the severity of disease, and the inspiratory time was set at 1 second. The PEEP value was selected based on one of two criteria (at the discretion of the researcher): the criterion associated with the highest peripheral oxygen saturation (SpO_2_) value, which was tested with a pulse oximeter, or the criterion associated with the best (high) dynamic compliance. The dynamic compliance was calculated as VT/(peak pressure - PEEP) and evaluated after a recruitment maneuver.

The same micro processed mechanical ventilator (Leistung PR4G, Córdoba, Argentina) was used throughout the study. The systolic blood pressure was measured using a noninvasive Doppler technique. The airway and esophageal pressure and the volume and flow delivered by the ventilator were recorded using a FluxMed Respiratory Monitor.

### Statistical analysis

Descriptive statistics, including median and interquartile range, were calculated.

We considered 31 to 43mmHg as the PaCO_2_ target value according to a previous publication.^(^^25^^)^

Kruskal-Wallis and Fisher exact tests were used to compare the continuous and discrete variables between groups.

Statistical software was used (Minitab 16 software, State College, USA). P values < 0.05 were considered statistically significant.

## RESULTS

Twenty healthy dogs (17 mixed breed, 2 Toy Poodles and 1 Rottweiler) and 10 critically ill animals (2 Toy Poodles, 2 Labrador Retriever, 1 Doberman, 1 Golden Retriever, 1 Pit Bull and 3 mixed breeds) were studied. The median weight was 15kg (IQR: 7 - 27kg).

**Figure 1:**
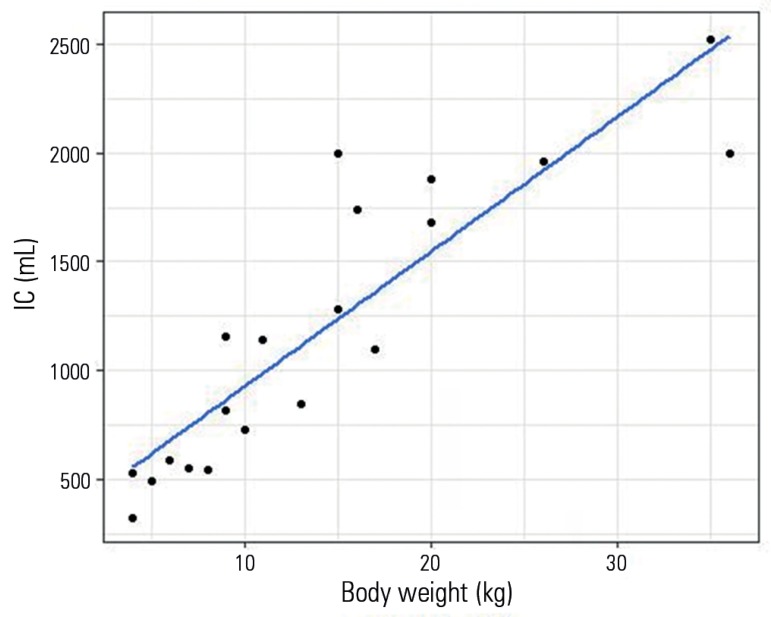
Relationship between body weight and inspiratory capacity in healthy dogs. IC - inspiratory capacity.

Figure 1 shows the relationship between IC and body weight in healthy dogs (Pearson's product moment correlation coefficient 0.889, p < 0.001). The median weight normalized IC was 84.67mL/kg (72.75 - 99.66).

The weight from healthy animals did not differ among VT groups (p = 0.962).

**Figure 2:**
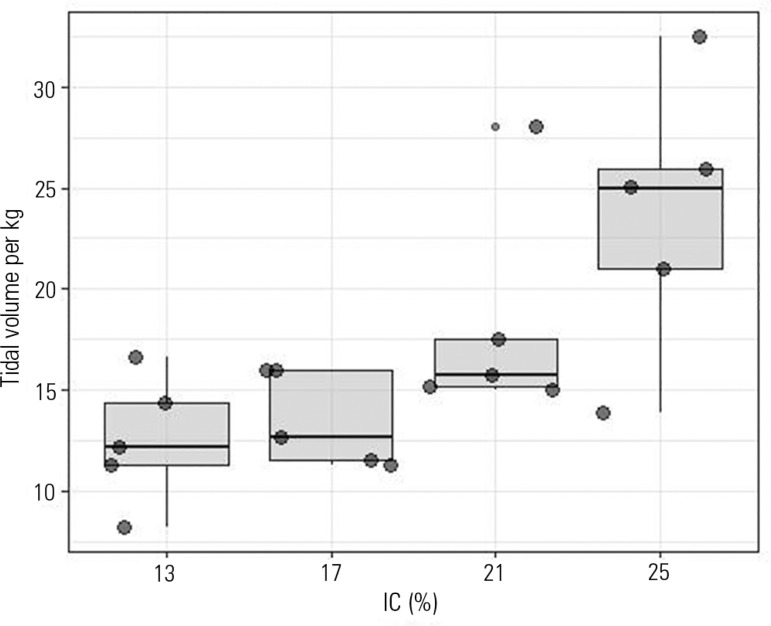
Tidal volume per kg of body weight according to inspiratory capacity percentage group. IC - inspiratory capacity.

The dogs of all groups displayed a broad range of tidal volumes per body weight (also, in some cases, retrieving identical VTs per body weight for different groups) when defined by different percentages of the IC (Figure 2).

**Figure 3:**
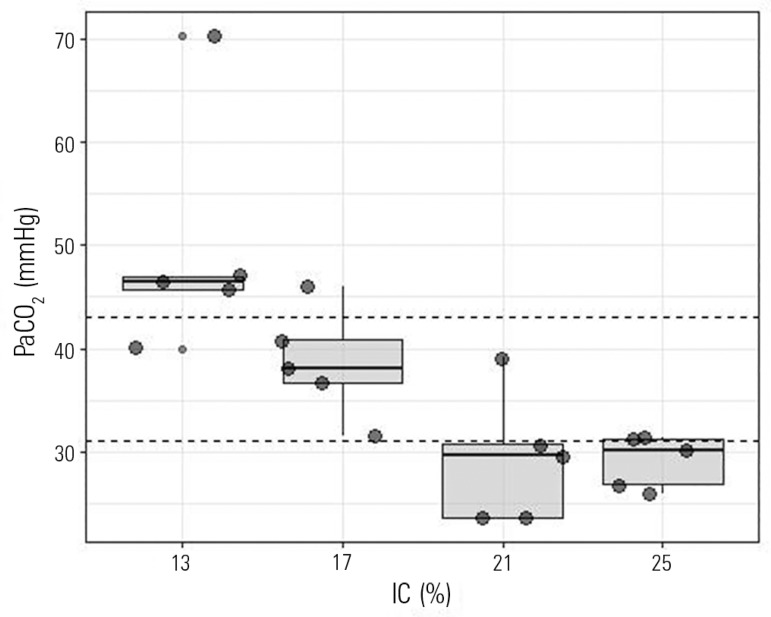
Partial pressure of carbon dioxide according to the inspiratory capacity percentage group. Horizontal dashed lines represent the expected partial pressure of carbon dioxide value. PaCO_2_ - partial pressure of carbon dioxide; IC - inspiratory capacity.

**Figure 4:**
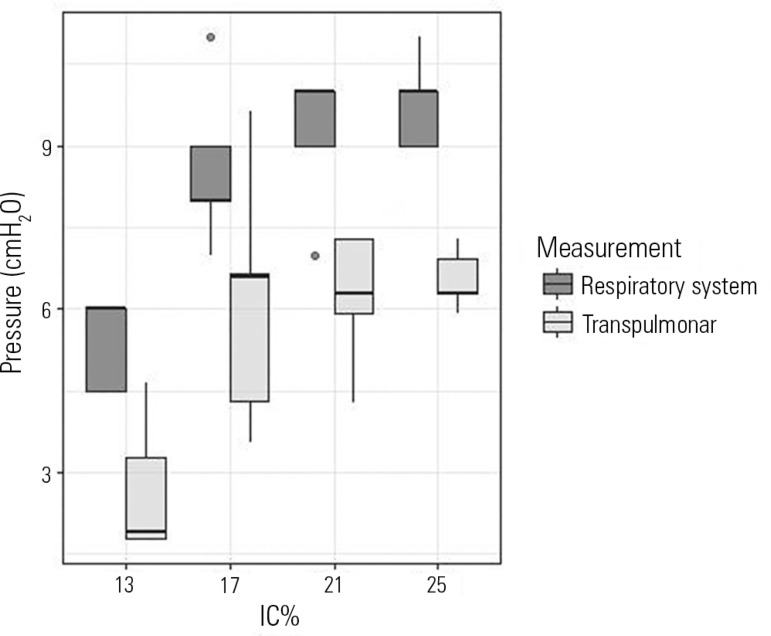
Static respiratory system and transpulmonary inspiratory pressures according to tidal volume group. IC - inspiratory capacity.

Table 1 displays the main physiological findings in healthy dogs. Healthy dogs ventilated with a VT of 17% of the IC were more frequently within the expected PaCO_2_ compared with the other groups (Figure 3 and Table 1). Figure 4 shows the inspiratory static respiratory system and transpulmonary pressures depending on the VT selection. Both static pressures were low even at higher volumes.

**Table 1:** Main findings in healthy dogs according to tidal volume group

	VT 13% of IC (n = 5)	VT 17% of IC (n = 5)	VT 21% of IC (n = 5)	VT 25% of IC (n = 5)	p value
Weight (kg)	15 (9 - 16)	13 (8 - 20)	10 (7 - 15)	11 (6 - 20)	0.962
Inspiratory capacity/weight	91.1 (85.33 - 108.75)	75.38 (67.5 - 94)	78.57 (73 - 80)	98.33 (84 - 103.63)	0.658
Tidal volume/weight (mL/kg)	12.2 (11.3 - 14.37)	12.69 (11.53 - 16)	15.71 (15.14 - 17.5)	25 (21 - 25.9)	0.037
Inspiratory peak pressure (cmH_2_O)	6 (5 - 7)	9 (8 - 9)	11 (10 - 11)	11 (10 - 11)	0.006
Inspiratory plateau pressure (cmH_2_O)	6 (4.5 - 6)	8 (8 - 9)	10 (9 - 10)	10 (9 - 10)	0.007
Inspiratory transpulmonary pressure (cmH_2_O)	1.92 (1.78 - 3.28)	6.6 (4.3 - 6.64)	6.28 (5.92 - 7.28)	6.28 (6.28 - 6.92)	0.026
PaCO_2_ (mmHg)	46.5 (45.7 - 47)	38 (36.7 - 40.8)	29.6 (23.6 - 30.7)	30.1 (26.8 - 31.3)	0.003
PaCO_2_ class (n(%))					0.004
High	4 (80)	1 (20)	0 (0)	0 (0)	
Low	0 (0)	0 (0)	4 (80)	3 (60)	
Target	1 (20)	4 (80)	1 (20)	2 (40)	

VT - tidal volume; IC - inspiratory capacity; PaCO_2_ - partial pressure of carbon dioxide.

To determine whether a VT selection using the IC measurement strategy results useful in pathological condition, we report the results of a series of ventilated critically ill dogs with a VT corresponding to 17% of the IC. Medical reasons indicating MV for critically ill dogs were profound shock (3 dogs), hypoventilation (2 dogs), respiratory failure (4 dogs) and profound shock with hypoventilation (1 dog). Table 2 summarizes the respiratory parameters of these animals after 10 minutes of MV. The median respiratory rate was 19 (15.5 - 20) bpm, and the initial FIO_2_ for all the dogs was 100%. Median PaCO_2_ was 34.45 (33.6 - 42.3) and was not significantly different from the values observed in healthy animals. Eight of 10 recorded PaCO_2_ values were within proposed ranges. The respiratory system and transpulmonary inspiratory pressures and driving pressure were significantly higher among the critically ill dogs (Table 3).

**Table 2:** Respiratory parameters of critically ill dogs

Dog	Age (years)	Weight (kg)	Inspiratory capacity (mL)	Tidal volume (mL)	Tidal volume (mL/kg)	Respiratory rate (bpm)	Inspiratory peak pressure (cmH_2_O)	Inspiratory plateau pressure (cmH_2_O)	PEEP (cmH_2_O)	PaCO_2_ (mmHg)
1	13	40	2400	410	10.25	20	15	11	3	43.6
2	8	6	480	80	13.33	25	15	12.28	3	35
3	10	4	380	65	16.25	18	10	7.3	0	40.2
4	6	27	1990	340	12.59	17	10	5.2	0	32.7
5	12	40	3380	570	14.25	15	12	9.28	2	33
6	10	40	2100	360	9	25	17	12.2	4	43
7	13	3	300	51	17	20	20	14.6	7	48.6
8	7	20	1760	299.2	14.96	15	10	NA	0	33.9
9	14	35	2090	355.3	10.15	15	9	NA	0	33.6
10	13	50	2580	438.6	8.77	20	13	NA	NA	33.6
Median (IQR)	11 (8.5 - 13)	31 (9.5 - 40)	2040 (800 - 2325)	347.65 (134.8 - 397.5)	12.96 (10.18 - 14.78)	19 (15.5 - 20)	12.5 (10 - 15)	11 (8.29 - 12.24)	2 (0 - 3)	34.45 (33.6 - 42.3)

PEEP - positive end-expiratory pressure; PaCO_2_ - partial pressure of carbon dioxide.

**Table 3:** Comparison of respiratory parameters between healthy and critically ill dogs ventilated with the same tidal volume selection strategy (17% of the inspiratory capacity)

	Healthy (n = 5)	Critically ill (n = 10)	p value
Weight (kg)	13 (8 - 20)	31 (9.5 - 40)	0.268
Inspiratory capacity/weight	75.38 (67.5 - 94)	76.85 (59.78 - 87.12)	0.594
Tidal volume/weight (mL/kg)	12.69 (11.53 - 16)	12.96 (10.18 - 14.78)	0.668
Inspiratory plateau pressure (cmH_2_O)	8 (8 - 9)	12.5 (10 - 15)	0.014
Driving pressure (cmH_2_O)	8 (8 - 9)	10 (10 - 12)	0.03
Inspiratory transpulmonary pressure (cmH_2_O)	6.6 (4.3 - 6.64)	11 (8.29 - 12.24)	0.048
PaCO_2_ (mmHg)	38 (36.7 - 40.8)	34.45 (33.6 - 42.3)	0.759

PaCO_2_ - partial pressure of carbon dioxide.

## DISCUSSION

The main finding of this study is that a simple strategy of VT selection for MV based on respiratory system measurements is useful in animals regardless of their lineage and size. The use of a VT based on IC is simple and requires only a few seconds. In fact, the only equipment required is a mechanical ventilator to measure volume and pressure. This approach may produce a better setting of ventilation compared with a more traditional method based on body size metrics. This may prevent hypoventilation and overstraining by adapting the parameters to the clinical conditions of the patient, considering the former situation as the main determinant of the impact of VT on the end inspiratory pressures.

Consistent with human physiology, during a maximal inspiration, the point at which respiratory system compliance starts to fall is near TLC. When referring to MV, this limit is marked by the upper inflection point of the static P-V curve, and its value is approximately 30cmH_2_O.^(^^26^^)^ Based on this concept, physicians recommend not exceeding this limit in critically ill human patients under MV.^(^^10^^,^^27^^)^ The TLC has been defined for healthy dogs as the lung volume during forced inflation at an airway pressure of 35cmH_2_O.^(^^4^^)^ This value is in line with our results. When generating the P-V curve, we found that the average upper inflection point corresponded to a value of approximately 35 cmH_2_O PiflexSupAw and 18cmH_2_O PiflexSupTP.

A VT setting for dogs ranging from 5 up to 20mL/kg of body weight was recommended.^(^^28^^-^^31^^)^ In a recent paper, Villar and Kacmarek state that all mammals, from an elephant to a shrew, have a VT that is related to body size at 6.3mL/kg, regardless of the body mass.^(^^32^^)^ In the same sense, allometric scaling studies proposed that the formula 7.69 x body weight^1,04^ (almost 8mL/kg body weight) best reflects the VT in all mammals.^(^^33^^)^ However, in an article published in 1972, Robinson et al. reported that the lung volume/body weight unit ratio is greater in dogs than in other species.^(^^34^^)^ The authors also suggested that the use of the same formula for all species is unlikely because of the heterogeneous anatomic structure of lungs in mammals, variation in thoracic conformations and the gravitational effects in the lungs of larger species. Additionally, in a study with 20 dogs of different breeds, the physiologic dead space had an average value of approximately 7mL/kg body weight.^(^^35^^)^ This is consistent with our findings, in which normocapnia at a VT of 12.69 (11.53 - 16) and 12.96 (10.18 - 14.78) mL/kg were observed both in healthy and critically ill dogs using the optimal IC based setting (17% of the IC).

In a recent experimental study in Beagles, researchers reported that the relationship between lung size (FRC) and body weight were not proportional in healthy dogs.^(^^36^^)^ This finding supports the hypothesis that the use of VT based on real body weight could be inadequate in this setting. The authors also developed a formula equivalent based on age, body size and weight that correlated well with the FRC. Unfortunately, this formula was not accurate in oleic acid injured dogs.

In our study, the variation in measurements in relation to the body weight among the healthy animals in our sample (72.75 - 99.66mL/kg) supports the hypothesis that the selection of VT based on actual lung size may be more suitable than selection based on body weight.

Driving pressure (DP) of the respiratory system (difference between inspiratory and expiratory static elastic pressures) has gained attention as a monitoring parameter to limit stress and strain in acute respiratory distress syndrome (ARDS).^(^^37^^)^ Because DP reflects the relationship between VT and compliance, the control of this parameter also allows adjusting the VT to the pulmonary functional size. For a given value of DP, the lower the compliance, the less the administered VT. Although it is currently recommended not to exceed 15cmH_2_O, there is not only one suggested DP value for MV setting. According to the strategy of selecting the VT based on the IC, a unique VT value can be obtained (the one that best fits the functional lung size), and the DP can be used for monitoring and eventually as an adjustment variable.

Recently, a review suggested that the transpulmonary driving pressure should be kept below 15 - 20cmH_2_O in patients with homogeneous lung parenchyma and possibly below 10 - 12cm/H_2_O in those with inhomogeneous parenchyma (ARDS).^(^^3^^)^ None of our healthy or critically ill dogs ventilated a VT equal to 17% of the IC, presented high inspiratory transpulmonary or static respiratory system pressures, and the driving pressures were low.

In the present study, the respiratory rate for healthy dogs was arbitrarily fixed at 15 bpm (a value that is in the middle of the range usually recommended for healthy dogs).^(^^28^^-^^31^^)^ This could be considered a limitation. According to some studies, small animals breathe faster than large animals.^(^^38^^)^ Allometric scaling studies^(^^33^^)^ proposed that the respiratory rate is represented by the formula 53.5 x weight^-0,33^. The use of a range for breaths per minute dependent on the size of the animal would have been more appropriate. Data suggest that faster rates should be used in small animals and slower rates in larger individuals.

Unfortunately, many variables were not monitored in this study. Volumetric capnography would allow a better understanding of the respiratory dead space and alveolar ventilation in each strategy. As MV may cause an important hemodynamic disturbance, blood pressure needs to be monitored. In the present report we applied non-invasive devices; however, invasive blood pressure monitoring would be a better option.

## CONCLUSIONS

In this study, we observed that inspiratory capacity normalized to body weight in healthy dogs from different breeds displayed significant variation. The selection of a tidal volume based on the body weight alone may result in over or under-strain in certain dogs. By contrast, tidal volume based on the inspiratory capacity of each animal is confirmed to be a useful and simple tool when setting ventilator parameters, both in healthy and critically ill dogs. In fact, this strategy allowed most of the dogs to achieve appropriate PaCO_2_ values and lung mechanics within acceptable physiological ranges. A similar approach should also be evaluated in other species including human beings if we consider the potential limitations of a tidal volume titration based on the calculated ideal body weight.

### Author contribution

PA Donati: Study design, data collection, analysis of data, literature search, manuscript preparation.

E Gogniat: Study design, data collection, analysis of data, literature search, review of manuscript.

M Madorno: Data collection, analysis of data.

EC Guillemi: Analysis of data, manuscript preparation, review of manuscript.

JM Guevara, MC Lavalle, FP Scorza and GF Mayer: Data collection, literature search.

PO Rodriguez: Study design, analysis of data, literature search, manuscript preparation, review of manuscript.
